# Gene-Editing Technologies and Applications in Legumes: Progress, Evolution, and Future Prospects

**DOI:** 10.3389/fgene.2022.859437

**Published:** 2022-06-28

**Authors:** Mehmet Cengiz Baloglu, Yasemin Celik Altunoglu, Pinar Baloglu, Ali Burak Yildiz, Nil Türkölmez, Yelda Özden Çiftçi

**Affiliations:** ^1^ Department of Genetics and Bioengineering, Faculty of Engineering and Architecture, Kastamonu University, Kastamonu, Turkey; ^2^ Research and Application Center, Kastamonu University, Kastamonu, Turkey; ^3^ Department of Molecular Biology and Genetics, Gebze Technical University, Kocaeli, Turkey; ^4^ Smart Agriculture Research and Application Center, Gebze Technical University, Kocaeli, Turkey

**Keywords:** genome-editing methods, CRISPR/Cas9, TALEN, ZFN, legumes

## Abstract

Legumes are rich in protein and phytochemicals and have provided a healthy diet for human beings for thousands of years. In recognition of the important role they play in human nutrition and agricultural production, the researchers have made great efforts to gain new genetic traits in legumes such as yield, stress tolerance, and nutritional quality. In recent years, the significant increase in genomic resources for legume plants has prepared the groundwork for applying cutting-edge breeding technologies, such as transgenic technologies, genome editing, and genomic selection for crop improvement. In addition to the different genome editing technologies including the CRISPR/Cas9-based genome editing system, this review article discusses the recent advances in plant-specific gene-editing methods, as well as problems and potential benefits associated with the improvement of legume crops with important agronomic properties. The genome editing technologies have been effectively used in different legume plants including model legumes like alfalfa and lotus, as well as crops like soybean, cowpea, and chickpea. We also discussed gene-editing methods used in legumes and the improvements of agronomic traits in model and recalcitrant legumes. Despite the immense opportunities genome editing can offer to the breeding of legumes, governmental regulatory restrictions present a major concern. In this context, the comparison of the regulatory framework of genome editing strategies in the European Union and the United States of America was also discussed. Gene-editing technologies have opened up new possibilities for the improvement of significant agronomic traits in legume breeding.

## 1 Introduction

Legumes are members of the angiosperm group and contain 19,500 species in 751 genera (Lewis et al., 2005). In addition to their nutritional value, the legume family contains many crops that contain essential amino acids and plant-based proteins. Additionally, legumes play an essential role in cultivating sustainable agriculture by symbiotically fixing nitrogen and releasing high-quality organic matter into the soil. Although legumes provide health benefits as well as ecological significance, their cultivation is affected by lower crop yields due to stress factors. The current focus is on accelerating genetic gains related to yield, stress tolerance, and nutritional quality. Most genetic improvement of legumes has been accomplished over the past half-century through pedigree and performance-based selection. To achieve faster genetic gains in legumes, novel genomics techniques and high-throughput phenomics are widely used and resulted in improved legume varieties that possess important agronomic traits (Varshney et al., 2018). To increase yield potential and reliability, different approaches have been used such as genomic selection (or marker-assisted selection) and precision breeding (gene editing) (Bhowmik et al., 2021). With increasing access to information on genes and haplotypes that contribute to agronomically significant traits, genome editing has allowed the modification of multiple SNPs without affecting the original characteristics of a popular cultivar. Genetic barriers such as ploidy differences prevent many legume crop species from exchanging genetic material naturally; so, the enormous genetic diversity they hold in their wild relatives, however, remains unused (Varshney et al., 2018). The availability of the complete genome sequence of organisms makes a significant contribution to the advancement of new-generation genome-editing studies. Compared to other family members of legumes, there are more new-generation genome-editing studies in *Lotus japonicus* (Sato et al., 2008), *Glycine max* (Schmutz et al., 2010), and *Medicago truncatula* (Young et al., 2011), all of which have been fully sequenced, which supports the importance of having a complete genome. Although the availability of the whole-genome sequences of other legume species includes common bean (Schmutz et al., 2014), mung bean (Kang et al., 2014), lentil (Bett et al., 2014; Bett et al., 2016), and pea (Kreplak et al., 2019), genome-editing trials for those plants have not been conducted.

This article presents the mechanisms of new-generation genome-editing technologies including TALEN (transcription activator-like effector nucleases), ZFN (zinc finger nuclease), and CRISPR/Cas9 [the clustered regularly interspaced short palindromic repeat (CRISPR)/CRISPR-associated protein 9 nuclease (Cas9)] systems, and detailed application examples of those technologies in legume family members. Moreover, the regulatory framework and future of genome-editing technologies in those crops have been extensively mentioned. This current study offers the comprehensive coverage of genome-editing studies in plants in the legume family, making it a collective resource.

## 2 Gene-Editing Technologies

### 2.1 CRISPR/Cas9

**FIGURE 1:**
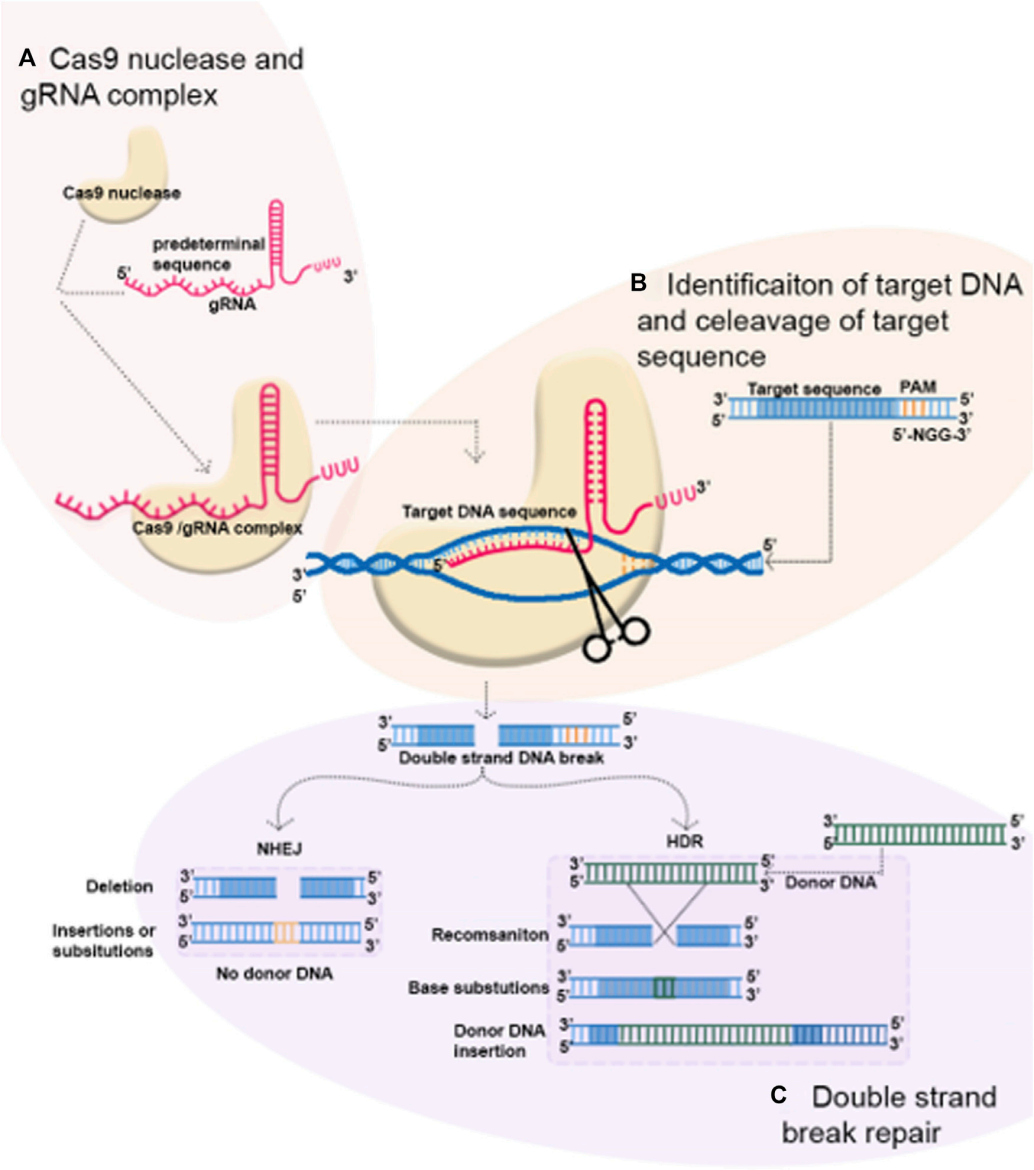
Mechanism of CRISPR-Cas9 genome editing. **(A)** CRISPR-Cas9 system is composed of the Cas9 protein and gRNA and Cas9/gRNA complex occur. **(B)** Cas9/gRNA complex cleave targets DNA in a binary complex, causing a double-stranded DNA break. **(C)** DNA breaks are repaired by non-homologous end joining (NHEJ) and homology-directed repair (HDR). In the process, short insertion deletions, nucleotide substitutions, or gene insertion may occur.

In recent years, CRISPR/Cas9 technology has become popular for genome editing in various organisms as well as in plants. It has also increased the scope of agricultural research by allowing the creation of novel plant varieties with the deletion of harmful features or the addition of prominent characters. CRISPR is a rapidly developing technique that can be used for a variety of genetic manipulations, including generating knockouts, making precise modifications, creating multiplex genome engineering, or activating and repressing genes (Arora and Narula, 2017). In the CRISPR/Cas9 system, there are two main elements: Cas9 protein and guide RNA (gRNA). Cas9 is an RNA-dependent DNA endonuclease that binds and makes a complex with gRNA (Figure 1A). The gRNA consists of 20 nucleotides that are complementary to the target DNA sequence and serves as a recruiting signal for Cas9. To recognize the target DNA sequence, CRISPR/Cas9 employs RNA–DNA interactions instead of DNA–protein interactions, which is the major difference between it and the other genome-editing technologies. Two different DNA-binding domains are needed for each target site for ZFNs and TALENs that employ DNA–protein interactions to target their specific sequences. This method is quite troublesome. When it comes to CRISPR/Cas9, which uses DNA–RNA interactions, only an 18–20 base pair needs to be designed. It is essential that Cas9 and gRNA attach to a specific protospacer adjacent motif (PAM), a sequence that is found at the 3′ end of target sequences (Figure 1B). The sequence 5′-NGG-3′ is the PAM for Cas9 from *Streptococcus pyogenes* that is the most frequently selected and utilized for genome editing. Through the introduction of double-stranded breaks (DSBs) in the DNA target, Cas9 induces DNA repair. The repair mechanism is achieved through non-homologous end joining (NHEJ) to make gene knockouts and homology-directed repair (HDR) to generate gene modification and gene insertion (Huang and Puchta, 2019) (Figure 1C). A frameshift mutation occurs when NHEJ randomly inserts or deletes a DNA strand within a coding region, resulting in a gene knockout. It is not necessary to use a homologous repair template for NHEJ. Therefore, the NHEJ repair mechanism is by far the most popular and optimized method of repairing DNA damage in all plants including legumes. The HDR technique, on the other hand, can exactly insert predetermined sequences coming from a donor DNA template. Due to the low editing efficiency of HDR, its application in plants has been restricted (Atkins and Voytas, 2020). With the CRISPR technology, potential applications include the analysis of gene expression, gain-of-function, and loss-of-function. Applications for CRISPR in legume farming have been performed, and this genome-editing technology can be used to produce high-quality, sustainable agricultural products, including legumes.

#### 2.1.1 Design of gRNAs

The gRNA, one of the main components of CRISPR/Cas9, plays a vital role in determining the specificity and efficiency of gene editing. Because of big gene families in plants, a high amount of sequence similarities and repetitive motifs occur and cause off-target gRNA binding, which is the main problem. As a result, gRNA binds unintended targets and unpredictable effects can be observed. To avoid these concerns, there are several requirements and preferences to take into account when selecting target motifs (Kumlehn et al., 2018). The most important limitation for choosing target DNA sequences arises from the availability of PAM that is triplet uniquely attached by the Cas endonuclease. In the case of Cas9 from *S. pyogenes*, PAM is composed of a flexible nucleotide followed by two guanosines, and this is known as NGG. To perform site-directed mutagenesis, the selection of two or three targets that are positioned 20 nucleotides upstream of a double G within the coding sequence of the target gene is adequate. The activity of Cas cleavage mainly depends on the secondary structure formation in the gRNA as there are requirements for the 5-terminal part of the gRNA to pair with the target DNA and the Cas endonuclease. Two-dimensional (2D) structures form inside the gRNA 3′ terminal scaffold and play a very important role in the function of gRNA (Ma et al., 2015). There are numerous online tools such as RNAfold (rna.tbi.univie.ac.at/cgibin/RNAWebSuite/RNAfold.cgi) and MFOLD (Zuker, 2003) available for the prediction of the secondary structure of gRNAs *in silico*. There are also some software programs designing gRNAs that create or demolish restriction enzymes following editing, allowing users to perform a quick screening of editing events. Some of them are listed below: For CRISPR-Cas nucleases, CRISPOR (Concordet and Haeussler, 2018), CRISPR-P (Liu et al., 2017), RGEN Cas designer (Park et al., 2015), and CHOPCHOP (Labun et al., 2019). For base editors, RGEN BE-Designer (Hwang et al., 2018) and PnB designer (Siegner et al., 2021). For prime editors, PrimeDesign (Hsu et al., 2021), pegFinder (Chow et al., 2021), and PlantPegDesigner (Lin et al., 2021). Despite the widespread use of software to design gRNAs, experienced users design gRNAs manually to suit specific purposes, such as detecting edits easily by restriction fragment length polymorphism analysis (Hassan et al., 2021).

#### 2.1.2 Selection of the Best Cas Protein

There has been an enormous increase in the number and varieties of CRISPR/Cas genome editing technology over the past 5 years. As of 2015, Cas proteins were categorized into five types and 16 subtypes, under two major Cas classes that differ profoundly based on the elements of their effector modules that process and interfere with gRNAs (Makarova et al., 2015). As genome engineering technologies have improved, type VI RNA-targeting and numerous types V CRISPR/Cas subtypes were developed to extend the class two system capabilities (Stella et al., 2017). Later, different versions of types IV, I, and V systems were identified to reside in mobile genetic elements because they lack the ability to cleave targeted DNA (Klompe et al., 2019). A recent study uncovered new proteins in the type II system that serves functions other than adaptive immunity (Niewoehner et al., 2017). Cas1, Cas2, Cas3, Cas5, Cas6, and Cas7 in type 1, Cas1, Cas2, Cas9, and Csn2/Cas4 in type 2, Cas1, Cas2, Cas6, and Cas10 in type 3, Cas5/Csf3, Cas7/Csf2, and Cas8/Csf1 in type 4, Cas12/Cpf1/C2c1 in Type 5, and Cas13/C2c2/CasRx in Type 6 are the samples of different CRISPR proteins. DNA nuclease, ribonuclease, RNA cleavage, and crRNA processing are their main functions (Makarova et al., 2020). Despite a large number of Cas proteins, only a small portion has been used in genome editing in plants. PAM restrictions, codon-optimization of Cas proteins, off-side effects, and temperature sensitivity are the main troubles to the selection of appropriate Cas proteins. Genome editing is mediated by various classes of CRISPR/Cas systems. Depending on their mode of genome editing, they can be divided into four categories: 1) point mutations, 2) deletions, 3) insertions, or 4) a combination of them. All these mutation modes were performed in different legume species, and the details of strategies are discussed in Section 3.

For genome editing of crops, the type II CRISPR/Cas9 system has been the most chosen. Because of the simple design of the CRISPR/Cas9 system, the availability of only one single gRNA and defined PAM sequences, Cas9 proteins are the most selected and studied proteins in genome-editing research in plants. Practically, the CRISPR/Cas9 system is designed for the replacement of the tracrRNA and crRNA molecules of the bacteria with the guide RNA (gRNA) (Cong et al., 2013; Mali et al., 2013). It became possible to simplify the system by containing just a gRNA to guide the Cas9 protein to the target and resulting in cleavage of the target region on DNA. Genome editing is accomplished by delivering both of these components to the nucleus. As with Cas9, Cas12 works on similar principles and in the same manner; however, Cas12 effectors prefer T*rich PAMs instead of G-rich PAMs of Cas9. This allows it to target specific genomic regions with greater effectiveness (Zetsche et al., 2015; Kim et al., 2017). The Cas12 systems do not require tracrRNA for maturation and interference. In contrast to Cas9 gRNAs, a single molecule of RNA engineered to a length of 44 nucleotides is utilized in Cas12 systems. Rather than blunt ends created by Cas9 effectors, Cas12 effectors cause double-strand breaks with staggered ends. This makes them ideal for targeting specific genes. Cas12a/Cpf1 systems that are isolated from *Francisella tularensis novicida* (FnCas12a), *Acidaminococcus* sp. BV3L6 (AsCas12a), and *Lachnospiraceae bacterium* (LbCas12a) are frequently selected for genome editing in different plant species with high success rates (Zhan et al., 2021). Cas13a has also been utilized for plant genome-editing studies along with Cas9 and Cas12. The system has a non-specific RNase activity and can exhibit the cleavage of ssRNA similarly to types II and V CRISPR systems (Abudayyeh et al., 2017). For this reason, it was suggested that RNA interference studies be replaced with this method. According to the literature, Cas9 protein is the most preferred and used for the development of genome-edited plants (Kiryushkin et al., 2022). The expression of Cas9 under the cauliflower mosaic virus (CaMV) 35S promoter (p35S) was observed in a total of 78 plant-related genome-editing studies (Amack and Antunes, 2020). To date, the CRISPR/Cas 9 platform has been mainly used in legume crops for the improvement of agronomic traits (see Section 3). It has been optimized for routine use in legume crops. Therefore, it is suggested that the Cas9 proteins can be more suitable for legumes.

#### 2.1.3 Vector Design

In recent years, CRISPR/Cas9—a genome-editing tool that has achieved worldwide fame—has been successfully used to edit the genomes of many monocot and dicot plants. To edit these genomes effectively, CRISPR/Cas9 components must be delivered to plants using an effective vector system that contains codon-optimized Cas9 gene and promoters for Cas9 and sgRNA. In addition, suitable target sites, efficient regeneration, and transformation methods must be specially optimized for the legume plants. It is necessary to deliver and express single guide RNA (sgRNA) and cas9 protein in the target cell for CRISPR editing (Arora and Narula, 2017). The expression of sgRNA is usually controlled by tissue-specific RNA polymerase III promoters such as AtU6 and TaU6. These promoters cause the production of specific small RNAs in various legume species. Like sgRNA, Cas9 has positioned downstream of RNA polymerase II promoters that guide the transcription of longer RNAs. For targeting nuclear DNA, Cas9 is mostly tagged with a nuclear localization sequence (NLS). The selection of suitable expression and Cas9 systems are critical factors for vector construction. Furthermore, restriction sites for the insertion of gRNA play a significant role. The website known as Addgene (http://www.addgene.org/crispr/plant/) provides information on the different types of plasmids for plant genome-editing studies. These plasmids in Addgene are empty backbones and usually possess three main components: sgRNA cassette, Cas9 endonuclease gene, and selection marker. RNA polymerase III promoters such as U3 or U6 have been obtained from monocot and dicot plants, and sgRNA has been directly expressed in plant cells. There are some genome-editing plasmids containing U3 or U6 promoters obtained from rice (Ma et al., 2015), maize (Qi et al., 2018), wheat (Xing et al., 2014), and Arabidopsis (Tsutsui and Higashiyama, 2016) and are commercially available in Addgene. In dicots and some monocots, a codon-optimized Cas9 under the control of the CaMV 35S promoter has been used. The maize ubiquitin promoter has been an alternative option to obtain homozygous, hemizygous, or biallelic mutations in the T_0_ generation that are passed on to subsequent generations (Shan et al., 2013; Zhang H. et al., 2014; Zhan et al., 2021). As the primary constituent of a CRISPR plasmid, the Cas endonuclease affects the rate of mutation during genome editing. SpCas9 (Cas9 from *S. pyogenes*) is the most preferred type of endonuclease used by researchers in plant genome-editing studies. For improvement of the performance of Cas9 endonuclease in the plant cell, different strategies have been developed. For example, codons of Cas9 have been optimized (Ma et al., 2015), the expression of Cas9 has been strengthened through strong promoters (Yue et al., 2020), and translational enhancers and nuclear localization signals have been added to the CRISPR cassette (Xie et al., 2015). For the efficient selection of genome-edited plants, different selection markers have been utilized. They are known as acetolactate synthase, phosphomannose isomerase, neomycin phosphotransferase, and hygromycin phosphotransferase (Hpt) (Yue et al., 2020). The *Hpt* gene, which confers tolerance to herbicide hygromycin, is the most frequently utilized marker selection gene in CRISPR-based plant breeding.

#### 2.1.4 Advanced Designs

Although HDR provides precise nucleotide substitutions in some plants including sugar cane (Oz et al., 2021), tomato (Vu et al., 2020), and maize (Svitashev et al., 2016), its application in plants including recalcitrant legume species such as lentils, soybean, chickpea, bean, and pea has limited because of low editing efficiency (Huang and Puchta, 2019; Atkins and Voytas, 2020). Considering these limitations in precise genome-editing technologies and the lengthy breeding process for legume species, agronomically significant properties may be achieved in much less time with new alternative CRISPR-based genome-editing tools for legumes. They are called deaminase-mediated base editing and reverse transcriptase-mediated prime editing, which are more efficient than HDR in plants. These new technologies do not require DSB formation and donor DNA. A:T > G:C and C:G > T:A transitions can be introduced directly into targeted sites by using adenosine deaminase (adenine base editor, ABE) and cytidine deaminase (cytosine base editor, CBE), respectively (Zhu et al., 2020). Next-generation sequencing technologies have led to the development of genome assemblies for a number of legume crops even if they are fragmented (Garg et al., 2021). These genomic information are used to modify key regions of genes in order to increase yield and quality, as well as other agronomic traits. Different studies discussed in this review indicated examples of the application of the classical CRISPR-based genome editing. All details related to the modification of legume genomes are displayed in Section 3 as a case study for each legume species. These studies may promote the fact that the modification of the complex nature of the legume genomes may also be defeated by these new base-editing and prime-editing CRISPR-based technologies. Utilization of the base-editing technology enables precise editing with high efficiency through both CBE and ABE systems. Various plant species including rice and tomato (Shimatani et al., 2017), rice, wheat, and maize (Zong et al., 2017), and wheat, rice, and potato (Zong et al., 2018) have been developed using different cytidine deaminase base-editing features. In recent studies, ABE systems have also been used in rice, wheat, *Arabidopsis thaliana,* and rapeseed (Kang et al., 2018; Li et al., 2018). In reviewing the literature, no data were found on the application of CBE and ABE systems for legume plants. Thus, various deaminase-mediated base-editing versions developed for model plant species may be useful for increasing the efficiency of the base editing in various legumes. Despite CBE and ABE systems having the ability to induct precise base transitions, their tools for base substitutions are restricted. Another highly promising technology known as “prime editor or prime editing” solves this problem by allowing the precise insertions of up to 44 bp, deletions of up to 80 bp, and combinations of these edits (Anzalone et al., 2019). The system has also been improved for plants and is able to perform multiple base substitutions, insertions, and deletions simultaneously in rice and wheat (Lin et al., 2020; Xu R. et al., 2020; Xu W. et al., 2020). Although different strategies and modifications such as the utilization of reverse transcriptase with different catalytic activities, the usage of ribozymes to obtain precise pegRNAs (prime-editing guide RNA), increasing culture temperature to raise catalytic activities, and modifications of the scaffold into pegRNA to augment the binding potential of Cas9 have been performed, there have been still limitations for the editing capacity of prime editor in plants (Zhu et al., 2020). Currently, the plant prime-editing technology has only been demonstrated in rice and wheat. Its performance still needs to be examined in a variety of plant species. Thus, plant prime-editing technology can be considered as an untouched deep blue cove for genome editing in legumes as well as in other significant agronomic plant species.

### 2.2 Transcription Activator-Like Effector Nucleases

**FIGURE 2:**
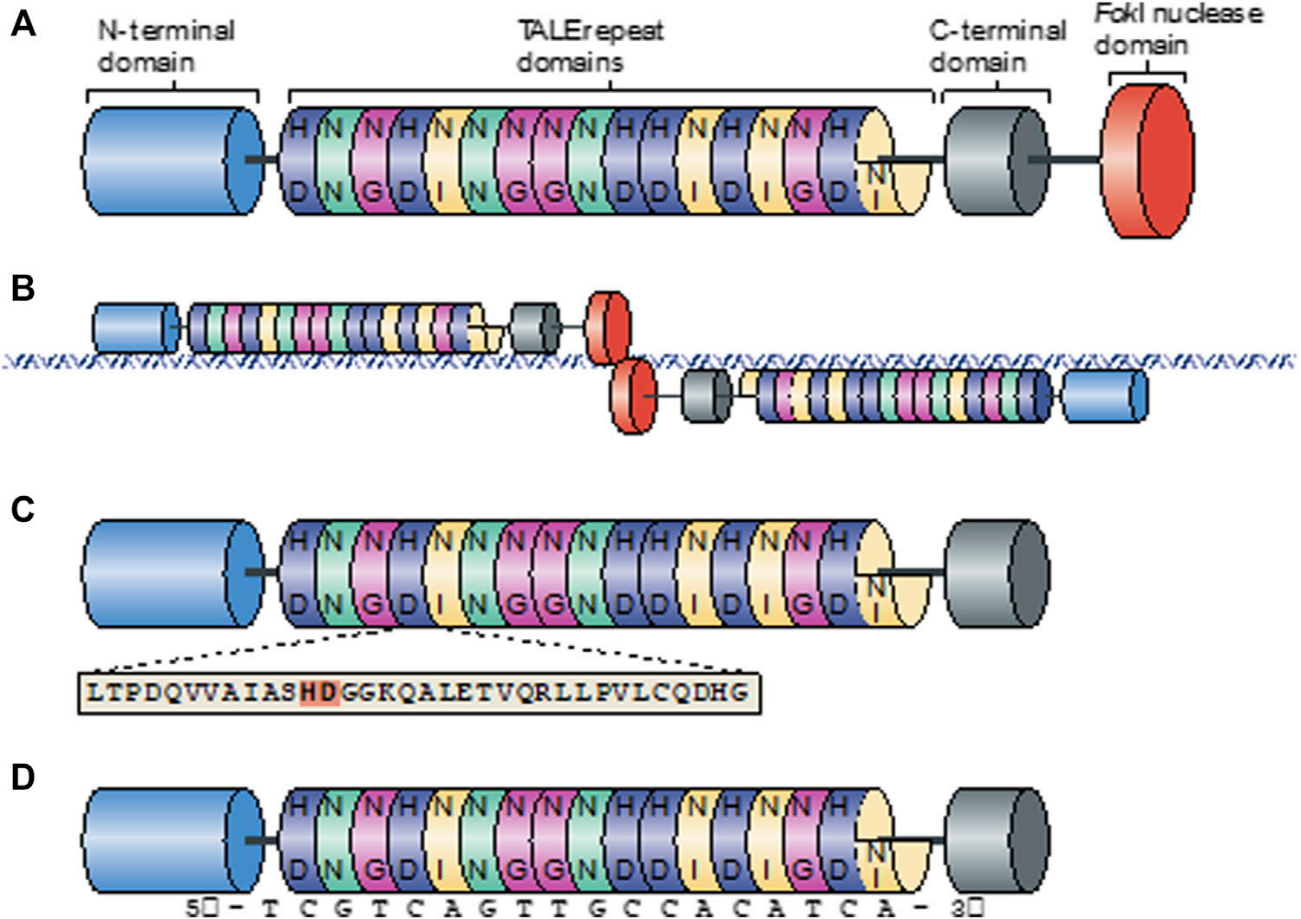
Overview of the TALEN structure (Joung and Sander, 2013). **(A)** Schematic view of the TALEN structure. Colored discs with two letters inside represent the RVD. **(B)** Schematic view of TALEN pair binding to target site. Cleave of Fokl occurs on the spacer domain on the target site. **(C)** Schematic diagram of the TALEN binding domain with an amino acid sequence. Two amino acids that represent RVD are shown in bold. **(D)** Amino acid sequence of a TALEN binding domain with a nucleotide representation of each RVD.

The origin of transcription activator-like effector nucleases (TALENs) is quite extraordinary. Like CRISPR, TALEN is derived from a bacterium, and interestingly, this bacterium, called Xanthomonas, is quite pathogenic and responsible for serious diseases in various crops. During the infection, with the type III secretion system, *Xanthomonas* translocates transcription activator-like (TAL) proteins into the host cell cytoplasm. To enhance bacterial colonization during the infection, TALs act as host’s-transcription factors and cause plant developmental changes that are beneficial for the disease. TALs mainly consist of three structures: the central domain of tandem repeats, transcriptional activation domain, and nuclear localization signals (Boch et al., 2009). Highly conserved repeat domains are mostly 33–35 amino acid lengths and are responsible for DNA binding. Specific target DNA to bind is decided by hypervariable residues that can be found at the 12th and 13th positions of the repeat domain. The pair of this position is named repeat-variable di-residue (RVD), and each RVD is associated with one of the four bases. On the other hand, in TALENs, *FokI* takes the place of the activator domain to become a target-specific genome-editing tool (Figure 2). To obtain double-strand breaks *via* FokI, TALENs are used in pairs. Each pair binds to the opposite strands of the target sequence and is separated with a spacer domain (Zhang X. et al., 2014). Before this groundbreaking development, *FokI* nuclease was placed along with the C-terminal activator domain on TALEN. Further studies have demonstrated that the truncation of large C-terminal sequences that are used to attend the *FokI* domain greatly increased the efficiency of TALEN (Bedell et al., 2012; Joung and Sander, 2013). These truncations were required due to the low efficiency of the custom TALEN application. Moreover, Bedell et al. (2012) aimed to increase the efficiency of TALEN by using differently truncated scaffolds. Their specifically designed GoldyTALEN scaffold, which is shorter due to the truncation of 215 amino acids from the pTAL scaffold, increased the success rate by up to sixfold, and some approaches showed 100% efficiency in zebrafish. All these results showed that design and construction have crucial roles and direct effects on the efficiency of the genome-editing process.

#### 2.2.1 Vector Construction

Since the construction of TALEN cassettes depends on many factors, researchers have invested considerable time and effort in this step to simplify the construction and increase the efficiency of TALENs. One of the most common molecular methods for the plasmid construction of TALENs is the golden gate assembly. This method enables the simultaneous assembly of multiple DNA fragments to a single plasmid. Since type IIS restriction enzymes take part in this method, multiple insertions to a plasmid could be done “scarlessly” as this type of restriction enzymes cuts DNA outside of recognition sites. Cermak et al. (2011) showed that with two steps it is possible to construct a vector with an array of 12–31 RVDs. The first step is for the assembly of RVDs into arrays, up to 10 RVDs each array, and the second step is for inserting these repeat arrays into the plasmid backbone. On the other hand, the assembly of 10 RVDs together is quite challenging. To eliminate this challenge during the cloning, Hegazy and Youns (2016) modified this protocol. Instead of 10 RVDs, they constructed five RVD length arrays. Even though this modification increased the duration of the construction, it also increased the rate of the successfully constructed plasmids from an average of 4.4%–30%.

### 2.3 ZFNs

The early 1990s were a critical turning point in the genome-editing perspective of view. With a better understanding of DNA repair systems, one of the first precise genome-editing techniques was developed. The so-called ZFN technique was developed by merging an engineered zinc finger domain with a nuclease domain (ZFN). Similar to TALEN, ZFs are combined with FokI for DNA cleavage and they are used as pairs to obtain double-strand breaks. On the other hand, the zinc finger domain consists of up to six proteins and is responsible for binding to the DNA target point. Engineered Cys_2_–His_2_ residues in the structures of these ZF proteins are stabilized by Zn^+2^ ions, and each one of these proteins interacts with three base pairs. For the construction, mainly three methods have been followed: 1) the modular assembly has been developed through the creation of identified ZF domain pool. During the application of ZFNs, the researchers can pick ZFs according to their target point from this pool and design their ZFN pair (Cathomen and Joung, 2008). 2) In context-sensitive selection strategies, the researchers have focused on developing new ZFN combinations of ZFs from customized libraries (Hurt et al., 2003; Durai et al., 2005). 3) The combination of two dual ZFs “(2 + 2 strategy)” from pre-existing libraries and optimization of these four ZFs according to the target loci with an algorithm. However, this method can only be used by researchers who have collaborated with Sangamo (Scott, 2005; Urnov et al., 2005).

Several advantages of CRISPR over ZFNs and TALENs have been pointed out in the literature, and the simplicity of the design has been highlighted as the main reason that made CRISPR the most widely utilized genome-editing tool. ZFN requires tremendous time and expertise during construction. Limited pairs have been identified so far, and optimization of zinc fingers is challenging. Especially protein engineering and the combination of new zinc fingers make this process impossible to perform in most of the laboratories. Although TALEN is much easier to construct when it is compared with ZFN, it is still far behind CRISPR. To construct a TALEN pair that targets a gene with 20-base pair length requires the design of 20 RVDs and the assembly of RVDs into a plasmid. These two steps make the process very challenging and again impossible to perform for researchers. Less than this effort, we can construct a CRISPR plasmid that targets 10 different genes in an organism. Even though it raises some questions, especially ethically inquiring questions, CRISPR became the most popular genome-editing tool due to its simplicity, efficiency, and multiplexed targeting potential. However, there are still some cases that make TALENs preferable. Due to the possibility of targeting longer DNA sequences, TALENs reduce the possibility of off-targets during the application. Researchers thus may choose TALENs over CRISPR to eliminate off-target mutations.

ZFNs, TALENs, and CRISPR-associated Cas9 endonucleases are the three major generations of genome-editing tools that have been mainly used for over a decade in plants including different legume species. The advantages and disadvantages of these methods have been discussed in many research articles (Gaj et al., 2013; Malzahn et al., 2017; Ahmar et al., 2020). Plant scientists face a major challenge in improving legume production and quality amidst changing climates and extreme environmental conditions. A promising option for achieving this goal is genome editing, and CRISPR/Cas technology is the most popular option because it is easy to use and convenient. However, no routine method has been proposed for the most efficient genome editing for legumes. The main reasons for this situation can be summarized as follows. The plantlet regeneration and genetic transformation of various legume species became a bottleneck for them. It is, therefore, necessary to develop an optimized protocol for the transformation and regeneration of legumes that is reproducible and reliable across species. In addition, there are some difficulties in CRISPR technology such as the on-target efficiency, off-target capacity, sgRNA design, and selection of proper Cas proteins. The next section summarizes all genetic modifications, including genetic transformation and genome editing, that have been done with legumes so far. At the end of the review, some suggestions are also presented to eliminate these problems.

## 3 Applications in Legumes

### 3.1 *M. truncatula* (Alfalfa)

*M. truncatula* is a model organism for legume crops because of its relatively easy transformation, short life cycle, self-fertility, diploidy nature, and small genome. Because of these features, it is widely studied in molecular and physiological research on legume crops. Michno et al. (2015) reported that they mutated soybean glutamine synthase (*GS1*) and chalcone-flavanone isomerase (*CHI20*) genes in *G. max* and β-glucuronidase (*GUS*) gene in *M. truncatula* by hairy root transformation (Table 1). Differently, Bottero et al. (2021) produced two transgenic alfalfa events (named as 3-1 and 5-1) by using CRISPR/Cas9 with the pBI121 binary vector containing the *GUS* gene and determined an average of 55% *GUS* inactivation. In the literature, many researchers prefer to study *phytoene desaturase* (*PDS*) genes because of its easy phenotypic observation to evaluate the success of an efficient CRISPR/Cas9 gene-editing tool. In 2017, Meng et al. (2017) developed an efficient CRISPR/Cas9 system for targeted *MtPDS* gene mutations in *M. truncatula,* and they observed that 32 of 309 T_0_ transgenic plants exhibited the albino phenotype. Sequencing analysis of randomly selected 16 transgenic plants from this 32 showed that all these albino plants carry mutations at the targeted site of the *MtPDS* gene. In addition, Wolabu et al. (2020) showed that UBQ10 promoter-driven Cas9 provides high mutation efficiency (95% in Arabidopsis and 70% in *M. truncatula*). Zhang et al. (2020) also targeted *MtPDS* genes using the CRISPR/Cas9 system, and all regenerated seedlings derived from the homozygous/biallelic *MtPDS* mutant showed albino phenotypes.

**TABLE 1:** Gene-editing technology in different legume crops.

Legume	Technique	Target (gene and function)	Result	References
*Medicago truncatula*	CRISPR/Cas 9	*MtSUP* (regulates the floral organ number)	MtSUP was found to be orthologous of *AtSUP*	Rodas et al. (2021)
	CRISPR/Cas 9	*CYP93E2* and *CYP72A61* (soyasapogenol B biosynthesis)	51 *CYP93E2* mutant plant lines	Confalonieri et al. (2021)
	CRISPR/Cas 9	*MtPDS* (coding phytoene dehydrogenase/chromoplastic protein)	70% mutation efficiency	Wolabu et al. (2020)
		*MtPDS* (coding phytoene dehydrogenase/chromoplastic protein)	Homozygous and biallelic mutants	Zhang et al. (2020)
	CRISPR/Cas 9	*NPD* genes (nodulation)	Smaller nodules, earlier onset of nodule senescence, ineffective nodules	Trujillo et al. (2019)
	CRISPR/Cas 9	*MtHen1* (*Hua enhancer1* gene)	Efficient mutation	Curtin et al. (2018)
	CRISPR/Cas 9	*FMO1-like*, *RFP1-like*, *ERDJ2*, *MEL1*, *PEN3-like*, *ACRE1*, *HLZ1-like*, *PHO2-like*, *PNO1-like*, *FBL1-like* (root and nodules)	Statistically significant effects on nodule production	Curtin et al. (2017)
	CRISPR/Cas 9	*MtPDS* (coding phytoene dehydrogenase/chromoplastic protein)	Albino plants	Meng et al. (2017)
	CRISPR/Cas 9	*GmGS1*, *GmCHI20*, *MtGUS*	Mutated genes	Michno et al. (2015)
*Lotus japonicus*	CRISPR/Cas 9	*Lbs* genes (nodule senescence)	Early nodule senescence	Wang et al. (2019)
	CRISPR/Cas 9	*CYP716A51* (triterpenoid production)	Non-production of triterpenoids	Suzuki et al. (2019)
	CRISPR/Cas 9	*LjCZF1* and *LjCZF2* (root nodule symbiosis)	Decrease in nodule formation	Cai, K., et al. (2018)
	CRISPR/Cas 9	*SNF* (symbiotic nitrogen fixation) genes	CRISPR/Cas9 system can effectively induce mutations in SNF related genes	Wang et al. (2016)
*Glycine max* (Soybean)	CRISPR/Cas 9	*GmPRR37* (photoperiodic flowering)	Changes in flowering time	Wang, L., et al. (2020)
	CRISPR/Cas 9	*GmLox1*, *GmLox2*, *GmLox3* (encoding lipoxygenases)	Loss of lipoxygenase activity	Wang, J., et al. (2020)
	CRISPR/Cas 9	*GmAGO7a* and *GmAGO7b* (controlling leaf pattern)	Inherited mutation until T_2_ lines	Zheng et al. (2020)
*Glycine max* (Soybean)	CRISPR/Cas 9	*GmFT2a* and *GmFT5a* (flowering time)	ft2a, ft5a, and ft2aft5a mutants	Cai et al. (2020)
	CRISPR/Cas 9	Pooled platform-102 candidate genes	Multiplex mutations	Bai et al. (2020)
	CRISPR/Cas 9	*KAS1* (conversion of sucrose to oil)	Deletion and insertion mutations	Virdi et al. (2020)
	CRISPR/Cas 9	*GmFAD2-1A*, *GmFAD2-2A* (biosynthesis of peakoil)	Increased oleic acid content	Wu et al. (2020)
	CRISPR/Cas 9	*GmSPL9* (plant architecture)	Altered plant architecture	Bao et al. (2019)
	CRISPR/Cas 9	*FAD2-2* (seed content improvement)	Increased oleic acid content	Al Amin et al. (2019)
	CRISPR/Cas 9	*Glyma03g36470* (eukaryotic translation initiation factor)	Insertion and deletion mutations	Di et al. (2019)
	CRISPR/Cas 9	*Glyma14g04180* (late-embryogenesis abundant protein)	Insertion and deletion mutations	Di et al. (2019)
	CRISPR/Cas 9	*Glyma06g136900* (uncharacterized protein)	Insertion and deletion mutations	Di et al. (2019)
	CRISPR/Cas 9	*GmFAD2-1A*, *GmFAD2-1B* (biosynthesis of peakoil)	Increased oleic acid content	Do et al. (2019)
	CRISPR/Cas 9	*Conglycinin* (*7S*) and *glycinin* (*11S*) (storage proteins)	Mutations in three of nine genes	Li et al. (2019)
	CRISPR/Cas 9	*E1* (flowering time)	Early flowering	Han et al. (2019)
	CRISPR/Cas 9	*GmDrb2a* and *GmDrb2b* (double-stranded RNA-binding2)	Biallelic double mutant	Curtin et al. (2018)
	TALEN	*Glycine max Dicer-like2* (Dicer-like protein)	*GmDicer-like2* mutant plants	Curtin et al. (2018)
	CRISPR/Cas 9	*GmFT2a* and *GmFT5a* (flowering time)	Deletion mutations	Cai, Y., et al. (2018)
	CRISPR/Cas 9 TALEN	*GmPDS11* and *GmPDS18* (coding phytoene dehydrogenase/chromoplastic protein)	Albino and dwarf buds	Du et al. (2016)
	CRISPR/Cas 9	*DD20* and *DD43* (two genomic sites on chromosome 4)	Mutations	Li et al. (2015)
	CRISPR/Cas 9	*Glyma06g14180* (uncharacterized protein)	Mutations	Sun et al. (2015)
	CRISPR/Cas 9	*Glyma08g02290* (potassium transporter)	Mutations	Sun et al. (2015)
	CRISPR/Cas 9	*Glyma12g37050* (ethylene receptor)	Mutations	Sun et al. (2015)
	CRISPR/Cas 9	*GmFEI2* (LRR receptor-like serine/threonine-protein kinase FEI 2)	Mutations	Cai et al. (2015)
	CRISPR/Cas 9	*GmSHR* (short root protein)	Mutations	Cai et al. (2015)
	TALEN	*FAD2-1A/B* (seed content improvement)	Increased oleic acid content, reduced linolenic acid content	Haun et al. (2014)
	TALEN	*FAD3A* (seed content improvement)	Reduced linolenic acid content	Demorest et al. (2016)
	TALEN	*GmDcl2b* (generating heritable mutations)	Combinatorial mutant plants	Curtin et al. (2017)
	ZFN	*RDR6a and RDR6b* (process optimization)	2 bp differences on target genes	Curtin et al. (2011)
	ZFN	*DCL4a/b* (Dicer-like protein)	Defective miRNA precursor transcript processing	Curtin et al., 2011, 2015
	ZFN	*DCLb* (Dicer-like protein)	Increased lateral root growth	Curtin et al. (2011)
Cowpea (*Vigna unguiculata*)	CRISPR/Cas 9	*VuSPO11-1* (cowpea meiosis gene)	Mutations	Che et al. (2021)
	CRISPR/Cas 9	*SPO11-1*, *REC8* and *OSD1* (meiosis genes)	Male and female sterilities	Juranic et al. (2020)
	CRISPR/Cas 9	*SNF* (symbiotic nitrogen fixation) genes	Blocked nodule formation	Ji et al. (2019)
Chickpea (*Cicer arietinum*)	CRISPR/Cas 9	*4CL* (*4-coumarate ligase*) *RVE7* (*Reveille 7*) (drought tolerance)	High efficiency in editing	Badhan et al. (2021)
Peanut (*Arachis hypogaea*)	CRISPR/Cas 9	*AhNFR1* and *AhNFR5* (nodulation)	Successfully edited genes	Shu et al. (2020)
	CRISPR/Cas 9	*AhFAD2* (seed content improvement)	G448A, 441_442insA, G451T mutations	Yuan et al. (2019)
	TALEN	*AhFAD2* (seed content improvement)	Increase in the oleic acid content	Wen et al. (2018)

*M. truncatula* forms indeterminate nodules, which are also found in pea (*Pisum sativum* L.), lentil (*Lens culinaris* Medik.), faba bean (*Vicia faba* L.), and chickpea (*Cicer arietinum* L.), that make it a good candidate plant to study nodulation in lentils (Bhowmik et al., 2021). In 2017, Curtin and colleagues used CRISPR/Cas9 nucleases, hairpin RNA interference constructs, and Tnt1 (the transposable element of *Nicotiana tabacum* cell type 1) retrotransposons together to evaluate the function of 10 candidate genes that exist in six clusters of strongly associated single nucleotide polymorphisms in *M. truncatula*. They found three candidate genes, ubiquitin conjugate24-like (*PHO-like*), Penetration3-like (*PEN3-like*)*,* and partner of NOB1-like (*PNO1-like*), having statistically significant influences on nodule production.

In 2019, Trujillo et al. (2019) identified nodule-specific polycystin-1, lipoxygenase, and alpha toxin nodule-specific (PLAT) domain proteins (NPDs) and examined the *NPD* function with its knockout lines *via* CRISPR/Cas9. They created different combinations of *NPD* gene inactivations and observed that mutant lines showed an earlier onset of nodule senescence and smaller or ineffective nodules in comparison to the wild-type control.

CRISPR/Cas9 gene-editing tool has also been used in studies focused on flowering or secondary metabolite production. Rodas et al. (2021) edited the *M. truncatula SUPERMAN* (*MtSUP*) gene with CRISPR/Cas 9 and determined the impairment of *MtSUP* function with observing defects in floral development and inflorescence architecture in *mtsup* mutant allel carrying plants. Confalonieri et al. (2021) also used the CRISPR/Cas9 gene-editing tool to knock out the two cytochrome P_450_ genes (*CYP93E2* and *CYP72A61*) that are responsible for soyasapogenol B production in *Medicago* spp. Their results showed that 51 putative *CYP93E2* mutant plant lines with an 84% editing efficiency did not produce soyasapogenols in the leaves, stems, and roots with diverting the metabolic pathway toward the production of valuable hemolytic sapogenins.

### 3.2 *L. japonicus* (Lotus)

*L. japonicus* is also a model organism for legume crops with similar features to *M. truncatula,* but conversely to it, *L. japonicus* organizes determinate nodules, like soybean [*G. max* (L.) Merr.] and cowpea (*Vigna unguiculata* L.). Wang and colleagues (2016) studied *L. japonicus* and proved that SNF (symbiotic nitrogen fixation)-related gene mutations can be performed by CRISPR/Cas9 with hairy root transformation. In 2018, Cai K. et al. (2018) edited cytokinin receptor Lotus histidine kinaz I-interacting protein (*LjCZF1*) to reveal the mechanism of cytokinin signaling regulation of rhizobia-legume symbiosis. They determined that the knock-out mutant lines had a significantly reduced number of infection threads and nodules, supporting that LjCZF1 is a positive regulator of symbiotic nodulation. Later, Wang et al. (2019) used CRISPR/Cas9 technology to understand the role of leghemoglobin (Lbs) in *L. japonicus* and they observed that the lack of Lbs resulted in an early nodule senescence. In another study, the gene loss-of-function analysis of *CYP716A51* (which shows triterpenoid C-28 oxidation activity) was performed and the results showed that cyp716a51-mutant *L. japonicus* hairy roots did not produce C28 oxidized triterpenoids.

### 3.3 *G. max* (Soybean)

There is an ever-increasing need for soybean products since soybean has an important economic value with its rich protein and oil for animal and human nutrition. For this reason, genetic development with gene-editing tools must be accelerated in order to meet this increasing need and cope with the changing environmental conditions (Bao et al., 2020). The initial optimization approach was done by Curtin et al. (2011) by targeting the green fluorescent protein (GFP) coding region in soybean with a ZFN array that was developed *via* context-sensitive selection strategies. This approach resulted in up to 71 base pair deletions on the target. With the optimization of the process, they targeted two different RNA-dependent polymerases in soybean. The most interesting outcome of this study was that two independent ZFN pairs were designed, both recognizing their specific targets and causing two base pair differences in both genes. Another study by the same group (Curtin et al., 2015) was focused on disturbing miRNA maturation and miRNA gene expression regulation in 2015. For this purpose, they designed two different ZFN pairs to target *Dicer-like 1a* (*DCL1a*) and *Dicer-like 1b* (*DCL1b*) genes in soybean. While single mutants of *DCL* genes did not give any remarkable result, double *DCL* mutants expressed remarkable morphological outcomes; additionally defective miRNA precursor transcript processing efficiency and deregulated miRNA target gene expression were observed. In addition, Curtin et al. (2018) also used TALENs within the *G. max Dicer-like2* gene. They revealed multiple transgene insertion events by whole-genome sequencing and generated a suite of combinatorial mutant plants.

In the case of CRISPR-Cas, Cai et al. (2015) targeted different sites of two endogenous soybean genes (*GmFEI2* and *GmSHR*). For this, they designed an sgRNA that targeted a transgene (bar) and six sgRNAs resulted in targeted DNA mutations in hairy roots. Li and co-workers (2015) successfully applied CRISPR/Cas9 and mutated two genomic sites DD20 and DD43 on chromosome 4 with 59% and 76%, respectively, success rates. Moreover, Sun and colleagues (2015) also constructed two vectors using the Arabidopsis U6-26 and soybean U6-10 promoters and targeted *Glyma06g14180*, *Glyma08g02290,* and *Glyma12g37050* in protoplast efficiently. In addition, *Glyma06g14180* and *Glyma08g02290* biallelic mutations were also observed in transgenic hairy roots. Later, Du et al. (2016) presented a comparative analysis of CRISPR/Cas9 and TAL-effector nuclease (TALEN) gene-editing technologies for two soybean *GmPDS11* and *GmPDS18* genes and they observed albino and dwarf buds (PDS knock-out) with the transformation of cotyledon nodes. The mutation efficiency of TALENs was slightly higher than the Cas9/sgRNA system using the AtU6-26 promoter but much lower when using the soybean GmU6-16g-1 promoter in hairy roots. According to the results, they declared that both gene-editing technologies can achieve gene targeting in soybean. In addition to this study, Curtin and friends (2018) also performed CRISPR/Cas9 and TALENs at the same time in *G. max* and *M. truncatula* and created a bi-allelic double mutant for the two soybean paralogous *double-stranded RNA-binding2* (*GmDrb2a* and *GmDrb2b*) genes and a mutation of the *M. truncatula Hua enhancer1* (*MtHen1*) gene.

Soybean flowering time is important due to its effect on increasing breeding speed for yield and improving quality. Because of this reason, many studies also focused on editing flowering time-related genes. Cai Y. et al. (2018) developed an efficient system using a dual-sgRNA/Cas9 to target deletions in *GmFT2a* and *GmFT5a* genes. Their results showed 15.6% and 15.8% deletion frequencies for target fragments in *GmFT2a* and *GmFT5a*, respectively*.* They also detected 12.1% exceeding 4.5 kb in *GmFT2a*. In addition, they determined that these deletions can be inherited in T_2_ “transgene-free” homozygous ft2a mutants that exhibited the late-flowering phenotype. In another study (Han et al., 2019), soybean maturity gene *E1*, which controls soybean flowering, was edited and 11 bp and 40 bp deletions at the *E1* coding region were generated. These deletions lead to premature translation termination codons and truncated E1 proteins. In addition, Wang L. et al. (2020) created knock-out and overexpression mutations with CRISPR/Cas9 tool in soybean *Pseudo-response regulator* gene (*GmPRR37*), encoding qFT12-2 (flowering time) protein and they demonstrated that *GmPRR37* controls soybean photoperiodic flowering. Cai et al. (2020) also studied *GmFT2a* and *GmFT5a* genes with CRISPR/Cas9 in soybean and showed that these genes collectively regulate flowering time by analyzing the overexpression of ft2a, ft5a, and ft2a/ft5a mutants under short-day SD and long-day conditions.

CRISPR/Cas9 strategy also used to target three GmLox genes (GmLox1, GmLox2, and GmLox3) encoding three lipoxygenases (LOX1, LOX2, and LOX3), which induce a beany flavor that restricts human consumption (Wang J. et al., 2020). They determined that 60 T_0_ positive transgenic plants, carrying combinations of sgRNAs and mutations (two of them triple mutant and one of them is a double mutant), had lost the corresponding lipoxygenase activities. Differently, Li et al. (2019) used the CRISPR/Cas9 system in editing *conglycinin* (7S) and glycinin (11S) storage protein genes in soybean and detected 5.8%, 3.8%, and 43.7% gene-editing efficiencies for *Glyma.20g148400*, *Glyma.03g163500,* and *Glyma.19g164900* genes, respectively. Besides, plant architecture is also altered by the application of CRISPR/Cas9 system in soybean. Bao et al. (2019) targeted squamosa promoter binding protein-like genes (*GmSPL9a*, *GmSPL9b*, *GmSPL9c,* and *GmSPL9*) and determined that T2 double homozygous mutant spl9a/spl9b has a shorter plastochron length. In addition, the increased node number on the main stem and branch number is observed in T4 mutant plants.

The cultivation of soybean varieties with higher oleic acid content becomes a major goal in breeding (Wu et al., 2020). In accordance, gene-editing technologies gained an increasingly important role in soybean studies. Although TALENs have not been widely used in legumes, there are a few successful TALEN applications, particularly to increase the oleic acid content and functional studies. Since the oleic acid content is dependent on the activity of *Fatty Acid Desaturase 2* genes, which are the key enzymes for converting oleic acid to linoleic acid that oxidizes readily, most of the studies were focused on introducing mutations at these genes. For instance, Haun et al. (2014) focused on increasing the soybean oleic acid content by targeting *FAD2-1A* and *FAD2-1B* genes. For targeting these two genes, four pairs of TALENs were designed. Only FAD2_T01 and FAD2_T04 were expressed by plants. The mutation rate of FAD2_T04 at both genes was 7.2%; on the other hand, the efficiency of FAD2_T01 was even lower than FAD2_T04, 3.1% at *FAD2-1A,* and 1% at *FAD2-1B*. A decrease in linoleic acid (down to 4%) together with an increase in oleic acid content (up to 80%) was obtained. A similar study was performed by Demorest et al. (2016), targeting *FAD2-1A*, *FAD2-1B,* and *FAD3A* genes. FAD3A_T1, FAD3A_T2, and FAD3A_T3 TALENs were designed to target the *FAD3A* gene of the FAD2-1A and FAD2-1B mutated lines, and they showed 11.2%, 16.0%, and 4.9% mutation rates, respectively. With these mutations, more than 80% increase in oleic acid and a reduction in decreased linoleic acid (2%) were obtained. Moreover, Do et al. (2019) targeted *GmFAD2-1A* and *GmFAD2-1B* genes and created T_0_ transgenic plants. The fatty acid profile analysis showed an 80% increase in the oleic acid content, whereas 1.3%–1.7% decrease in linoleic acid in T_1_ seeds homozygous for both *GmFAD2* genes. Similarly, Al Amin et al. (2019) applied the CRISPR-Cas9 system for the mutation of the *FAD2-2* gene in soybean and observed an important level change in oleic acid/linoleic acid ratios caused by high-frequency deletions and insertions in the gene. In 2020, Wu and co-workers also used CRISPR/Cas9 in *GmFAD2-1A* and *GmFAD2-2A* genes to create single and double knock-out mutants and showed that their function was slightly changed. Their editing efficiency was determined as 95% for *GmFAD2-1A*, 55.56% for *GmFAD2-2A,* and 66.67% for double mutants. They also determined that the oleic acid content increased up to 73.50%, while the linoleic acid content decreased down to 12.23% in the T_2_ generation. In addition, these contents showed similar level changes in the T_3_ generation.

Di et al. (2019) enhanced the CRISPR/Cas9 system by using highly active 5 U6 promoters by targeting *Glyma03g36470*, *Glyma14g04180,* and *Glyma06g136900* genes. Results showed that nucleotide insertion, deletion, and substitution mutations occurred. The following year, Bai et al. (2020) constructed 70 CRISPR-Cas9 vectors to target 102 candidate genes and they obtained 407 T_0_ mutant lines containing all sgRNAs with 59.2% mutagenesis frequency. In addition to this, 35.6% of lines carried multiplex mutations. As a result, increased nodule numbers in gmric1/gmric2 double mutants and decreased nodulation in gmrdn1-1/1-2/1-3 triple mutant lines were observed.

Zheng et al. (2020) presented easy-to-use binary vector systems with Cas9 driven by egg cell-specific promoters (ECp). They targeted two genes, *GmAGO7a* and *GmAGO7b*, coding ARGONAUTE7 (AGO7), which are key regulators in controlling leaf patterns in soybean. Their results showed that these promoters can induce mutations and multiple, independent mutations can be obtained. In another study, Virdi et al. (2020) generated multiple knockout alleles and also one in-frame allele for the *β-ketoacyl synthase 1* (*KASI*) gene, which has a role in changing sucrose to oil, by using CRISPR/Cas9 mutagenesis and their results indicated that genes lost their function.

Due to the importance of soybean, relatively more CRISPR studies including the modification of its nutrition value and plant architecture (leaf patterns and nodule numbers) were carried out in soybean among other legumes. However, the stable soybean genetic transformation has not yet been established since the soybean is still a recalcitrant crop to transformation. With the improvement of the transformation efficiency, CRISPR studies could advance future genetic studies in soybean with its efficiency, multiplex editing, and high-throughput mutagenesis capability (Bao et al., 2020).

### 3.4 *V. unguiculata* (Cowpea)

Cowpea (*V. unguiculata* (L.) Walp.) is a legume crop that has a high nutrition content and health benefits. It has an efficient symbiotic nitrogen fixation (SNF) capability, tolerance to low rainfall, and low fertilization requirements. Due to these agronomically important traits, it became one of the most important legumes worldwide (Ji et al., 2019; Che et al., 2021). For these reasons, interest in gene-editing approaches in cowpea is increasing. In 2019, Ji and colleagues demonstrated CRISPR/Cas9-mediated genome editing in cowpea in non-inheritable mutated hairy roots by targeting SNF genes. They observed that nodule formation was completely blocked in the mutants with both alleles disrupted. Following them, Juranic et al. (2020) identified three cowpea meiosis genes; *SPO11-1* (encodes SPO11 protein, which is the initiator of meiotic double-stranded breaks), *REC8* (encodes meiotic recombination protein), and *OSD1* (encodes *Ophiostoma scytalone* dehydratase protein that promotes meiotic progression) used CRISPR/Cas9 gene editing to induce asexual seed formation in cowpea. They determined biallelic mutations in exon 1 and exon 3 of the *SPO11-1* gene resulting in defects in meiosis leading to complete male and female sterilities in the T_0_ plants. Recently, Che et al. (2021) also knocked out the cowpea meiosis gene *VuSPO11-1* by using CRISPR/Cas9 and observed mutations at the target.

### 3.5 *C. arietinum* (Chickpea)

Chickpea (*C. arietinum*) is a commercially important crop worldwide, and gene-editing tools can be used to eliminate the problems in its production. Badhan et al. (2021) performed a study that targeted drought tolerance-associated genes, *4-coumarate ligase* (*4CL*) and *Reveille 7* (*RVE7*), for CRISPR/Cas9 editing in chickpea protoplast and the knock-out of the *RVE7* gene showed high-efficiency editing *in vivo*. These results showed that CRISPR/Cas9 DNA-free gene editing can be used for genes associated with drought tolerance in chickpea by utilizing protoplast. To date, this was the first and only study that used CRISPR/Cas9 gene editing in chickpea.

### 3.6 *A. hypogaea* (Peanut)

Peanut (*A. hypogaea*) is an important legume crop with a high oleic acid content. A high oleate spontaneous mutant line (F435), which contains 80% oleic acid, has previously been identified by plant breeders *via* a peanut germplasm screening project (Norden et al., 1987). In this line, two types of mutations were reported in the *ahFAD2A* [a “G” to “A” substitution at 448  bp after the start codon (G448A) in the ahFAD2A] and *ahFAD2B* [an “A” insertion between bp 441 and 442 (441_442insA) in the ahFAD2B] genes (Lopez et al., 2000). Yuan et al. (2019) targeted these mutations and in addition, they observed a new mutation, G451T, in *ahFAD2B*. These results suggested that the mutations induced in *ahFAD2B* by CRISPR/Cas9 may be useful in developing high oleate lines. Moreover, TALENs are also used to create targeted *ahFAD2* genes in peanut to increase the oleic acid content (Wen et al., 2018). Two TALEN pairs were constructed, one of them was used to inoculate 216 regenerated roots and the second one was used to inoculate 105 regenerated roots. Observed mutation frequencies were 8.33% and 12.38%, respectively. Most of the mutations occurred as small deletions of 1–10 bps. In the mutant lines, the oleic acid content of seeds was determined as 80.45%, which means a 2-fold increase when compared to wild-type plants. On the other hand, the linoleic acid content was decreased down to 3% in the mutant lines and there was no change in the total fatty acid amount.

Nod factor receptors (NFRs) that initiate a symbiotic relationship with rhizobia also edited with CRISPR/Cas9 to reveal out their functions in peanut nodulation (Shu et al., 2020). The edited mutants with two *AhNFR5* genes showed Nod-phenotype, while two selected *AhNFR1* genes containing mutants still could form nodules after inoculation.

### 3.7 Non-edited Legume Species

Lentil (*L. culinaris*) is a diploid and a self-pollinated plant in the Fabaceae family, containing rich proteins, minerals, fibers, and carbohydrate sources. Among developing countries and those whose diets are not based on expensive animal protein, it can contribute to the prevention of malnutrition and deficiencies in micronutrients (Kumar et al., 2015). Besides, as lentils add nitrogen to the soil, the quality of the soil improves (Kumar et al., 2021). A draft lentil whole genome sequence is available at https://knowpulse.usask.ca/lentil-genome, which contains bulk sequencing, gene prediction, and annotation of the assembled 2.6 Gbp of the genome (Bett et al., 2014; Bett et al., 2016). The available draft genome sequences can facilitate the sequence-based targeted candidate genes related to nutrient value, abiotic and biotic stress responses, herbicide resistance, etc. Genomic tools and technologies can help to improve lentil breeding. Some gene transformation efforts are available for lentils. As a successful example of those studies, the *dehydration-responsive element binding* gene (*DREB1A*), which is involved in plant responses to abiotic stresses, was transferred to lentils by *Agrobacterium*, resulting in drought- and salinity-resistant transgenic plants (Khatib et al., 2011). *In vitro* regeneration after transformation is another important issue. It was reported that using decapitated embryos than other tissues was more effective in the generation of shoots (Sarker et al., 2003). Those attempts supply useful tools for new genome-editing research. Many candidate genes related to abiotic, biotic stress factors, and agronomic features have been determined in lentils (Kumar et al., 2021). However, up to date, there is no new genome-editing research studying candidate genes in lentils using ZFNs, TALENs, or CRISPR/Cas9 technologies. Genome-editing technology can be an easy and cheap way to discover the function of those candidate genes to provide cultivars with desired features including stress tolerance or agronomic traits (Bhowmik et al., 2021).

Pea (*P. sativum*) is an important legume crop in the world after the common bean (*Phaseolus vulgaris*) and has rich components including dietary proteins, mineral nutrients, complex starch, and fibers (Bastianelli et al., 1998). Pea’s symbiotic nitrogen-fixing ability makes it a valuable crop for the development of systems that improve soil fertility (Mabrouk et al., 2018). In addition, the pea is an original model organism used by Mendel to construct the rules of inheritance (Ellis et al., 2011). The pea genome size is approximately 4.45 Gb. A reference genome was published in 2019, which provides insights into legume genome evolution (Kreplak et al., 2019). The genomic approach has an essential role in determining genes for critical features and developing genomic tools for crop improvement. Although significant progress has been made in pea planting, improving crop yield and quality, crop development must continue to feed the growing world population.

Peas are affected by parasitic weeds, viruses, bacteria, and fungi as much as abiotic stress factors including drought, salinity, heat, and cold stresses, which result in the loss of yield and growth. A stable transformation study was successfully applied in pea transferring *cry1Ac* gene (encoding protoxin) from *Bacillus thuringiensis* (Negawo et al., 2013) and alpha-amylase inhibitor gene from *P. vulgaris* (Schroeder et al., 1995) for insect tolerance *via Agrobacterium*-mediated transformation method. Another transformation attempt was applied for transferring antifungal genes against *Fusarium* spp. to pea, which resulted in a lack of stable expression in 3 years of field trials (Kahlon et al., 2017). *Agrobacterium*-mediated transformation efficiency and regeneration frequency were enhanced from seed-to-seed regeneration using longer infection time and adding zeatin to the selection medium in a recent study (Aftabi et al., 2017). The available genome sequence information, successful transformation, and regeneration applications are factors that affect the usage of genome-editing tools in vegetable crops (Cardi et al., 2017). To date, no studies were conducted using new genome-editing tools in pea, which may be attributed to the insufficient regeneration (Pandey et al., 2021). The development of new genome-editing methodologies can provide new opportunities in breeding to increase yield and produce plants with high nutritional value.

Common bean (*P. vulgaris*) is the most used up grain legume around the world, which has rich nutritious elements including proteins, vitamins, and minerals (Cichy et al., 2009). The common bean whole genome sequence has been available since 2014. In addition, researchers determined genes related to improved leaf and seed mass in common bean (Schmutz et al., 2014). Although successful gene transformation studies are limited in common bean, many indirect and direct gene transfer attempts have been done (reviewed by Nadeem et al. (2021)). One of those attempts involved the construction of transgenic lines that display tolerance to golden mosaic geminivirus (BgMV-BR) *via* transferring *Rep-TrAP-REn* and *BC1* genes to common bean by the biolistic method (Aragão et al., 1998). Xue et al. (2017) transferred the *PvPOX1* gene from a *Fusarium* wilt-resistant genotype to a *Fusarium* wilt-susceptible genotype by *Agrobacterium rhizogenes*. Moreover, variable protocols were applied to improve the regeneration performance of the plant as other legumes (Nadeem et al., 2021). Accordingly, Barraza et al. (2015) reported that the manipulation of *PvTRX1h* gene, which is the ortholog of a plant histone lysine methyltransferase involved in plant hormone synthesis, can help to overcome recalcitrant regeneration problems in the plant as it regulates somatic embryogenesis in common bean callus. Researchers have also tried to improve the tolerance of common bean to major biotic diseases (white mold, bacterial blight, rust, halo blight, anthracnose, and pests) and abiotic stresses. Besides, some other features in the common beans including higher content of minerals (iron and zinc), fast cooking time, canning quality, harvest index, and market class/seed color are significant breeding preferences (Assefa et al., 2019). Although the availability of the whole genome information, CRISPR/Cas, or other genome-editing tools has not been utilized up to now in common bean genetic research, gene-editing technologies can be applied to common bean research to obtain stress-tolerant plants and to meet common bean breeding priorities.

Faba beans have growing advantages over other legumes in cold temperatures; therefore, they are suitable for sustainable farming applications (Temesgen et al., 2015). Furthermore, like other legumes, faba beans have valuable systems to raise soil fertility. Faba bean breeding maintains the need for food and feed, which has a valuable source of protein, fiber, and other nutrients (Khazaei et al., 2021). Publicly available genome sequence data of faba bean are not available, which can be the result of the hardness of assembling the huge genome (Cooper et al., 2017). Faba bean is one of the legume species that transformation and regeneration efficiency are mostly hard although the availability of the attempts obtained by *Agrobacterium*-mediated transformation with low transformation efficiency (Cardi et al., 2017; Maalouf et al., 2019). One of these attempts was firstly made by Böttinger et al. (2001) who used *in vitro* development of internode stem segments invaded by *Agrobacterium* and by Hanafy et al. (2005) who cut out embryo axes infiltrated by *Agrobacterium* to obtain stable transgenic lines. Moreover, abiotic stress-tolerant lines were reported by Hanafy et al., 2013 by transforming the potato *PR10a* gene to faba bean by the same transformation method. Many biotic and abiotic stress factors including heat, insects, viruses, and parasitic weeds cause decreased faba bean yields (Cernay et al., 2015; Maalouf et al., 2019). TALEN technology, which is one of the genome-editing tools, has been applied to construct disease tolerance in plants. It was achieved by upregulating resistance gene expression *via* an engineered promoter site, which can bind multiple TALL effectors (Romer et al., 2009) or by changed TALL binding regions of promoters in sugar transporter genes, which are targeted by pathogens to stimulate tolerance in rice (Li et al., 2012; Xiao, 2012). Another advantageous method, namely CRISPR/Cas9, can be used to gain plant virus resistance by destructing the viral genome or by modifying the plant genome for resistance (Lenka et al., 2020). Moreover, vicine and convicine limiting faba bean consumption were defined as pyrimidine glycosides in cotyledons of faba bean, which are toxic effects for humans with a mutation in the enzyme of glucose-6-phosphate dehydrogenase (Luzzatto and Arese, 2018; Khazaei et al., 2021). Those compound levels can be controlled using new genome-editing tools in faba bean.

Mung bean (*Vigna radiata*) is a rapidly growing legume in warm climates in the Fabaceae family. Mung bean has rich dietary protein, folate, and iron in the seeds compared to other legume species (Keatinge et al., 2011). Also, mung bean plants can keep atmospheric nitrogen, which provides improved soil fertility (Maalouf et al., 2019). The whole genome sequence of *V. radiata* var. *sublobata* published in 2014 has enabled genomic research and molecular breeding of mung bean (Kang et al., 2014). However, limited mungbean germplasm and incompatibility with wild relative species affect mung bean breeding (Aasim et al., 2019). In addition, biotic and abiotic stresses also reduce mung bean production. Therefore, progress in transformation technologies supports researchers to develop lines that can cope with abiotic and biotic stress factors. Although genetic transformation efficiency was low in mung bean, many studies *via Agrobacterium-*mediated transformation are available for different agronomic features such as insecticidal (Saini et al., 2007), oxidative stress tolerance (Yadav et al., 2012), and salt stress tolerance (Sahoo et al., 2016). In those studies, the selection of the explants, transformation vectors, and selective agents are the significant factors that affect the success of the transformation efficiency (reviewed by Tripathi et al. (2021)). Although there are no studies using new genome-editing tools in mung bean, the availability of the whole genome sequence and improvement of the *in vitro* regeneration and transformation procedures can enable the usage of those more sophisticated genome-editing tools to obtain crops with desirable features.

The availability of whole-genome data on the most common non-edited legume species including lentil, pea, common bean, and mung bean, which have abundant nutrients for the human diet worldwide, may allow genome-editing approaches to be developed. Identification and demonstration of the functions of genes related to abiotic and biotic stress tolerance, yield and quality, etc., represent a broad application area for new genome-editing tools to improve those characteristics in the important legume crops that are valuable for human nutrition. Furthermore, legumes are indispensable to soil fertility, so symbiotic nitrogen-fixing pathways could prove useful in genome-editing applications that aim to improve soil quality. Although there have been some promising attempts, the low transformation efficiency and the recalcitrant regeneration problems in those legumes appear to be the most significant limitations, which may be one of the common reasons for the lack of new genome-editing attempts. In order to realize the substantial improvement promised by new genome-editing techniques in those legumes with serious potential, new approaches to improve transformation and regeneration efficiency are critical for the adequate feeding of a large part of the world.

## 4 Future Prospects

### 4.1 Future of Genome-Editing Technologies in Legumes

Crop breeding and functional genomics have progressed rapidly with genome-editing research including the Nobel prize awarded system, CRISPR. Recent advancements in genome-editing research have increased the accuracy and efficiency of modifying genes by adding or removing the genetic material. Besides, genome-editing technologies have been performed to facilitate the manipulation of single or multiple genetic loci in different plants. The recently sequenced variable legume genomes are a valuable source of information for researchers with better applications of gene-editing tools. Therefore, a thorough understanding of legume genomic sequences and their functions is essential for efficient genome editing. It is possible to develop new legume varieties with the identification of genes that control certain traits in legumes, such as taste, size, disease resistance, and drought tolerance.

Gene-editing technology is an efficient, precise, and crucial way of meeting the health needs of an increasingly populous world and helping farmers cultivate better crops. In addition to agricultural implications, CRISPR technology could be used more widely in the future to clarify genomic structures and their role in all plants and legumes. For instance, CRISPR technology may improve the understanding of transcriptional regulation of Cas9 and Cpf1, the monitoring of genetic loci and mechanisms, and the regulation of promoter activity in plants (Ahmar et al., 2020). Furthermore, it will cover single-nucleotide polymorphisms (SNPs), as well as genome-wide association studies to change and better understand the epigenetic behavior of legumes. By creating a genome-wide association study in *M. truncatula,* this technique was able to identify the nodulation-associated genes (Curtin et al., 2017) and also the mutational research of five different nodule-related PLAT domains (NPD1-5), and member genes of the nitrate peptide family (NPD) have been identified (Trujillo et al., 2019; Wang, L., et al., 2020).

Hairy root transformation is a long been used technique that enables the production of transgenic roots in a quick and straightforward manner. It is mainly chosen when there are no protocols existing for stable transformation and regeneration, or the desired traits were only seen in roots (Christey, 2001). Recently, a study described the possibility of editing the genomes of transgenic hairy roots using CRISPR/Cas9 (Kiryushkin et al., 2022). This study concluded that by combining these techniques, it is possible to study gene function quickly and efficiently. Therefore, Hairy CRISPR, the term used in Kiryushkin’s study, can be considered as another alternative application of genome engineering tools to overcome genome-editing problems in recalcitrant legume species.

**FIGURE 3:**
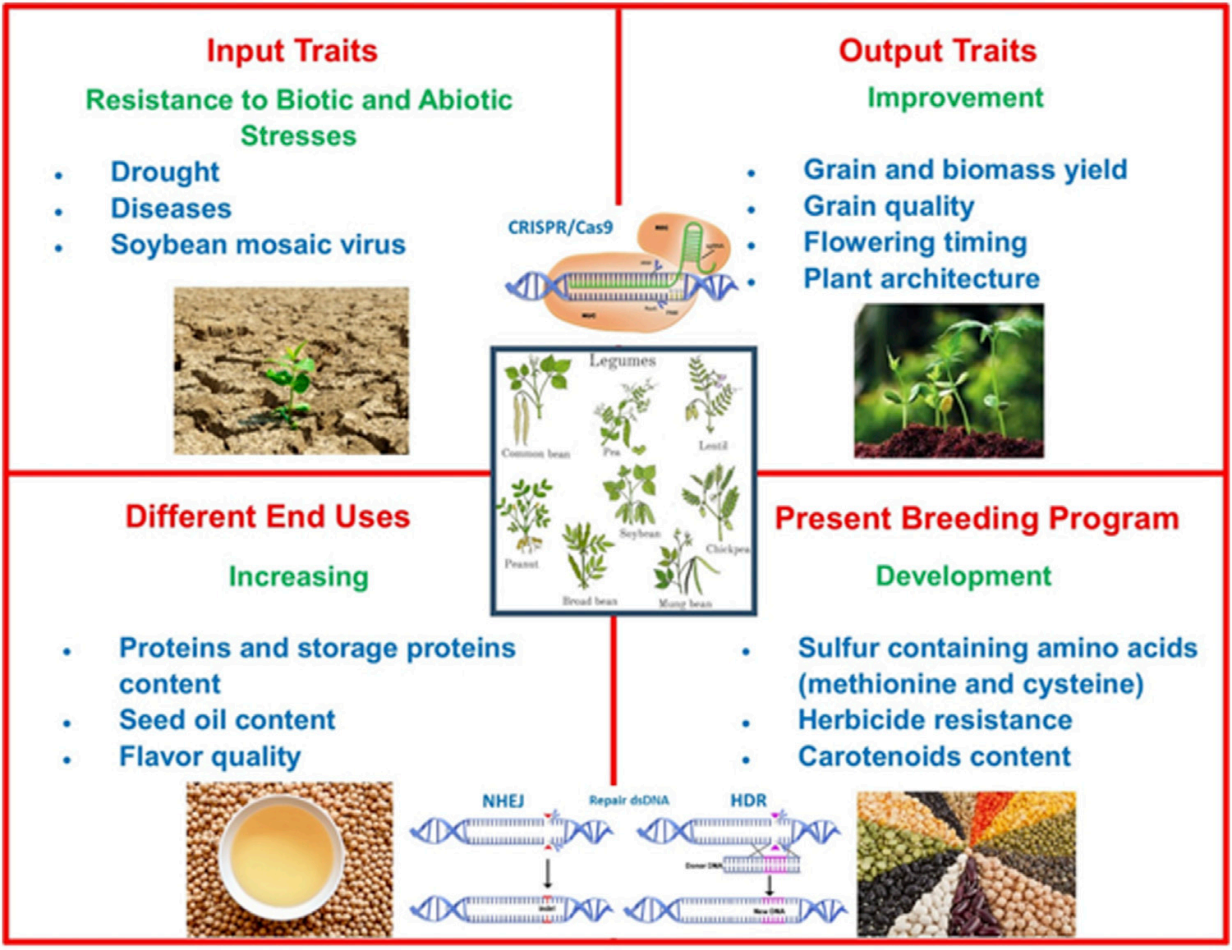
Strategies for improving legume breeding.

CRISPR/Cas9 utilization in legume breeding programs can be implemented in the future to improve different prominent agronomically important features including biotic and abiotic stress resistance, quality and nutritional value increase, augmentation of carotenoid content, and obtaining sulfur-containing amino acids (Figure 3). Novel studies discussed in this review show that genome-editing technologies including CRISPR/Cas9 have been widely used for gaining significant traits for legumes, but it is still needed to improve efficient regeneration and transformation systems, reliable screening and selection strategies, and construction of multi-purpose vector systems.

To overcome these problems, several approaches that could be used in near future are summarized as follows:

-Since legumes still have low regenerative capacity, *de novo* meristem induction (Maher et al., 2020) could be used to eliminate tissue culture steps.
-To improve transformation efficiency, new methods could be developed, including the delivery of transformation vectors into germline cells (Zaidi et al., 2020).
-Using inducible promoters (i.e., heat-inducible CRISPR) instead of constitutively active promoters could also help to increase the efficiency of gene targeting in legumes, as it worked well in maize (Barone et al., 2020).
-CRISPR still needs to be improved to reduce off-targeting for its extensive use in legumes.
-Although pea is still not gene-edited, its eIF4E virus-resistance allele (eIF4E1)-encoded N176K substitution with base-editing of the Arabidopsis *eIF4E1* gene generated clover yellow vein virus (ClYVV)-resistant Arabidopsis plants (Bastet et al., 2019). Biomimicking of this natural polymorphism that existed in legumes could be used for the induction of biotic stress tolerance in crops as well as other legumes.
-Even though CRISPR eliminates the possibility of the presence of foreign DNA in the final product, extended field trials should also be carried out to see the performance of the GE plants prior to their commercialization.

### 4.2 Regulatory Framework of Genome-Edited Legume Crops

In this section, genome-editing application and regulatory framework to all plants were examined in detail, since there is no specific regulation for genome-edited legumes. Both public and private breeders believe that gene editing, the latest innovation in genetic engineering, has great potential to develop new plant varieties. It is possible to edit the genome in several ways resulting in different products: allele replacement, site-directed deletions, site-directed insertions (or site-directed nucleases-SDN-1/2/3 in the terminology of Friedrichs et al. (2019a)), and base conversion (Marzec and Hensel, 2020). CRISPR/Cas9 genome-editing system, one of the most widely used genome-editing systems, has gained wide-scale adoption for its application in biomedicine, agriculture, industrial biotechnology, and other bioeconomy sectors (Friedrichs et al., 2019b). According to the jurisdiction, each of these may be subject to a different regulatory approach. Plant breeders might need to meet different requirements for research, legal, regulatory, and marketing when developing new genome-edited plants using those “genome-editing techniques” (Entine et al., 2021). In many countries, gene-editing regulation initially caused a great deal of confusion, which has been cleared up in the past 4 years. The advent of genome editing has brought new regulatory challenges, particularly in relation to regulatory differences and traceability, which can lead to new types of obligations and trade dilemmas. Across many countries and regions in the world, different regulatory approaches were examined in this section.

GE/GM organisms are regulated in Australia, New Zealand, Europe, and India through a process-driven regulatory trigger. These jurisdictions are revising the content of their regulatory definitions to reveal whether all kinds of genome editing are covered under their existing GE/GM regulatory frameworks. The current situation has shown that all plant varieties produced using the gene-editing technology would have to meet the same standards as GMOs as required by the European Union (EU). A Technical Review of the Gene Regulations had been started by the Office of the Gene Technology Regulator (OGTR) for Australia in October 2016, which resulted in some proposed changes. According to these new regulations, GMO regulations would not apply to organisms modified with site-directed nucleases without templates for genome repair (i.e., SDN-1). As with organisms modified by oligonucleotide-directed mutagenesis, organisms modified using a template to direct genome repair (e.g., SDN-2, SDN-3, etc.) are GMOs. There is still an ongoing discussion among regulatory agencies regarding the regulation of all new technologies according to the existing regulatory framework in India.

GE/GM and genome-editing products are regulated in Canada and the United States according to a product trigger, under which the novelty of a particular trait is evaluated on a case-by-case basis, regardless of the technology used to develop it. Leader in the production of GM crops, the United States has proclaimed that any crop variety containing no foreign genes would be regarded as a conventionally bred crop variety rather than a GM crop. A risk-based, product-triggered regulatory approach is followed in Canada. Biotechnology products derived from gene editing in Canada are subject to a pre-market safety assessment only if they are new (i.e., display a new characteristic) and could pose new risks. Gene-edited products do not need pre-market safety assessments in Canada if they do not exhibit a novel trait (i.e., “novel” refers to “new to the Canadian environment or to the food or feed supply in Canada”). Argentina was one of the first countries to adopt a regulatory solution for new (plant) breeding techniques (N(P)BTs) in 2015, covering the genome-editing subcategory as part of it. If the NBT contains no new genetic material, a non-GM regulatory classification is implemented.

Consequently, genome editing is regulated in different ways in different countries. Because of country-specific regulations, genome-editing regulations are not harmonized globally. Moreover, a variety of regulatory and policy approaches to genome editing need to be understood by different jurisdictions. Although uniform global approaches are not possible, a common understanding is essential for reducing the troubles arising from the use of different regulations. There are many potential applications of genome-editing technology, from medicine and healthcare to food and agricultural production that could help address many of the grand challenges facing the 21st century society. Therefore, market acceptance of genome editing, as well as a transparent discussion of both risks and benefits, will be crucial to any governance activity. Due to the complexity of genome-editing technology, regulators and risk assessors must update their knowledge to respond to escalating information requirements. For genome editing to become a marketable technology, all stakeholders need to prioritize both communication and information exchange. Both advocates and opponents should explain risks based on science without sensationalizing or scaring the public.

## 5 Conclusion

Emmanuelle Charpentier and Jennifer Doudna were awarded the 2020 Nobel prize in Chemistry due to the usage of the world’s most popular genetic engineering tools, CRISPR/Cas, in medicine, agriculture, and food industries. Although CRISPR technology has more time for routine use in legumes, it is clear that this new-generation genome-editing technology will make important contributions to legume breeding studies to raise productivity and to improve biotic and abiotic stress tolerance with the improvement of technical (i.e., regeneration and transformation) capability of the legumes together with a reduction of off-targets, generation of multiple PAM site selection system, development of tissue-culture free protocols, enhancement of HDR and viral vector efficiencies for CRISPR, and regulatory and policy environment.
